# The Use of Situational Judgement Tests for Paramedic Selection in Canada: A National Cross-Sectional Survey

**DOI:** 10.7759/cureus.116175

**Published:** 2026-09-13

**Authors:** Ryan G Tingley, Jan L Jensen, Judah Goldstein, Aaron Sibley

**Affiliations:** 1 Paramedicine, University of Prince Edward Island, Charlottetown, CAN; 2 Paramedicine, Ambulance New Brunswick, Medavie Health Services, Fredericton, CAN; 3 Emergency Medicine, Dalhousie University, Halifax, CAN; 4 Health and Wellness, Nova Scotia Health, Halifax, CAN; 5 Emergency Medicine, Nova Scotia Health, Halifax, CAN; 6 Operations, Emergency Health Services, Dartmouth, CAN; 7 Emergency Medicine, Dalhousie University, Charlottetown, CAN

**Keywords:** continuing education, multiple mini-interviews, non-technical skills, paramedic, selection, situational judgment tests, survey

## Abstract

Background

It is important for paramedics to have strong interpersonal and cognitive skills. Despite the importance of such skills, the paramedic field has incorporated few tools to evaluate these attributes. Situational Judgment Tests (SJTs) are psychometric tools that use scenario-based questions to quantify personal and cognitive attributes. Though extensive literature exists on the use of SJTs, few studies explore the current state of SJT use in the paramedic field.

Methods

In this national survey study, we aimed to understand the current use of SJTs and other tools in the hiring of frontline paramedics by Canadian paramedic services. The survey also included questions about the use of Multiple Mini Interview (MMI), which are station-based interviews that have been found to validly evaluate the interpersonal and cognitive skills of paramedics for the purpose of hiring decisions.

Results

A total of 21 services left unique, complete responses. Neither SJTs nor MMIs are widely used in Canada. Only smaller paramedic services reported that they were using SJTs and MMIs, despite these tools being designed for mass testing.

Conclusions

Though a role has been identified for SJTs in paramedic selection and education, paramedic services in Canada do not appear to widely use SJTs to inform their hiring processes.

## Introduction

Situational Judgment Tests (SJTs) are psychometric tools that use scenario-based questions to quantify personal and cognitive attributes [1]. These tests can be delivered in written, verbal, or digital forms and are useful in large, high-stakes selection processes such as student applications to professional schools or the hiring of employees into high-pressure fields [2]. The validity and utility of SJTs have been studied in medicine, pharmacy, and education, and SJTs are now widely used for selection in these fields [3-5]. The anonymous format of written SJTs compared to traditional, interview-based selection methods may reduce assessor bias, as SJTs perform well in measures of demographic fairness [2]. The construct validity of SJTs, their ability to measure what they intend to measure, can vary greatly based on how the SJT is developed [6].

The United States government used scenario-based personality tests in the early 20th century to psychometrically evaluate soldiers [7]. Inspired by these tests, Motwildo, Dunnette, and Carter developed “low-fidelity simulation” in 1990, using written scenarios to predict subjects’ job performance [8]. Motwildo, Dunnette, and Carter [8] speculated that the detached nature of their test had allowed them to predict broader personal and cognitive traits; this was the jumping-off point for modern SJT research.

Bennett and Williams first introduced SJTs into the paramedic literature in 2019 and have identified a potential role for SJTs as a reusable evaluator of non-technical skills (NTS), which are personal or cognitive attributes that contribute to job performance [9,10]. Despite this, Bennett and Williams’ recommendation to construct a paramedic-specific SJT and assess its validity has not been fulfilled. Written SJTs are, in theory, highly cost-effective due to their ability to be reused infinitely if kept confidential [1]. The potential for reuse also enables SJTs to be used for continuing education through the identification of “weaker” aspects of respondents’ personalities, which can then be "strengthened"; it would be difficult to accomplish this with an interview-based tool, due to the amount of resources it would consume [10].

However, interview-based selection methods are in no way obsolete. The Multiple Mini Interview (MMI) is a series of rapid interviews that each intend to measure a specific personality trait. MMIs have been shown to validly evaluate NTS in the paramedic context as well as the medical context, and may quantify personality traits more accurately than SJTs [11,12]. Both of these selection tools are worth considering when developing selection processes; each presents a unique set of benefits.

This survey primarily sought to provide data on how widely used SJTs are by paramedic services in Canada for screening applicant candidates into paramedic positions. Secondary objectives were to provide insight into how these SJTs were being developed, what barriers may be preventing the further implementation of SJTs, and to provide parallel information on MMI use.

## Materials and methods

Ethics

The research ethics board at the University of Prince Edward Island (UPEI) reviewed this study and provided a waiver of full review.

Design

This national cross-sectional online survey of paramedic services organizations was designed and distributed using the online software LimeSurvey (LimeSurvey GmbH, Hamburg, Germany) [13]. The Consensus-Based Reporting of Survey Studies (CROSS) checklist was used to report results on survey development and implementation [14]. 

Recruitment and distribution

An email survey invitation was distributed to 81 member paramedic services of the Paramedic Chiefs of Canada (PCC), with a subsequent reminder email. The email and first page of the survey included background, ethical considerations, and instructed that the survey be completed by someone involved in the development of the hiring process, and be completed with the hiring of front-line paramedics in mind. Participants could click a link in the email, consent to participate in the study, and then complete the survey. This link was the only way to access the survey. Internet Protocol (IP) addresses were tracked to eliminate duplicates, and services were asked to self-identify, though this was not mandatory. The survey opened on December 19, 2024, and closed on February 26, 2025. 

Survey tool

Authors RT, JJ, and AS developed the survey together. The survey was pilot tested with 18 UPEI students to evaluate flow and comprehension during the week of December 9, 2024. The final survey contained 32 questions, although only about half of these could normally be answered per submission due to the survey logic. Only the first question was mandatory.

First, the survey asked which assessment tools were used in the hiring process of the responding service. The answer to this question determined much of the logic for the rest of the survey. If services did not use SJTs or MMIs, they were asked five questions on the development and implementation of their hiring process. Services that used SJTs were asked eight questions on the development and implementation of their SJT, and services using MMIs were asked 10 questions on the development and implementation of their MMI. Two questions related to barriers were posed to respondents who did not use SJTs, and another two were posed to respondents who did not use MMIs. Respondents who did not use SJTs or MMIs could answer all four barrier questions. Whether they were answering a question about SJTs or MMIs, respondents were given the same eight barrier options with the wording changed slightly. The survey ended with four questions to determine the geographic location of the service and the number of paramedic employees. The entire survey tool can be viewed in the Appendices. 

Data analysis

Descriptive statistics were used to characterize service characteristics. Survey responses were analyzed and reported as a proportion of the total paramedic service response. Responses were only included if at least six questions were answered. Duplicate responses were identified using demographic questions and IP addresses, and then the response with the fewest questions answered was eliminated. If services gave duplicate answers with the same number of questions answered, the more recent response was eliminated. No personal information was collected from respondents.

## Results

Response rate

We received 37 responses from the 81 services represented by the PCC. We eliminated six duplicate responses and 10 incomplete responses; 21 responses were included in the analysis (25.9%). The location and service size questions were not completed in five submissions. 

Characteristics of responding paramedic services

Survey respondents were from eight Canadian provinces and territories (Table 1). Over half of the responses were from Alberta (five, 29.4%), Ontario (four, 23.5%), and Saskatchewan (three, 17.6%). New Brunswick, Quebec, Nunavut, the Northwest Territories, and the Yukon were not represented by the survey; each of the other provinces was represented once. British Columbia, Nova Scotia, and Prince Edward Island have a provincial paramedic service; therefore, only one response would be expected for these provinces. Responding services varied in size; services employing under 200 paramedics were most common (seven, 43.8%), while large services with over 1500 paramedic employees were infrequent (two, 12.5%). Services were most commonly hiring under 30 paramedics per year (seven, 43.8%). Management (16, 76.2%) and human resources (12, 57.1%) were involved in the development of most of the hiring processes.

**Table 1:** Characteristics of 21 Paramedic Services Demographic characteristics of all services who provided complete answers to our survey. *Respondents could select multiple answers.

Characteristic	n (%)
Paramedic employees (n = 16)	
Less than 200	7 (43.8%)
200-600	3 (18.8%)
600-1500	4 (25.0%)
1500+	2 (12.5%)
Paramedics hired in the past year (n = 16)	
Under 30	7 (43.8%)
30-80	4 (25.0%)
80-150	2 (12.5%)
150+	3 (18.8%)
Province of service (n = 17)	
Alberta	5 (29.4%)
Ontario	4 (23.5%)
Saskatchewan	3 (17.6%)
Newfoundland and Labrador	1 (5.8%)
Nova Scotia	1 (5.8%)
British Columbia	1 (5.8%)
Prince Edward Island	1 (5.8%)
Manitoba	1 (5.8%)
Screening tools used* (n = 21)	
Interviews	17 (81.0%)
Clinical Scenario Test	11 (52.4%)
Written Clinical Test	4 (19.0%)
Multiple Mini Interviews	4 (19.0%)
Situational Judgment Test	3 (14.3%)
Other	6 (28.6%)
Developers of hiring process* (n = 21)	
Management	16 (76.2%)
Human resources	12 (57.1%)
Clinical training department	8 (38.1%)
Internal focus group	2 (9.5%)
External consultants	2 (9.5%)
Unsure	1 (4.8%)
Other	4 (19.0%)

Characteristics of respondents using SJTs or MMIs

Three services (14.3%) reported using SJTs as part of their hiring process, and four (19%) services indicated they used MMIs. No service reported using both a written SJT and an MMI or included SJT-style questions as a part of their MMI (Table 2). Each SJT had been developed by management (three, 100%) with the help of outside resources. Management (four, 100%) and human resources (four, 100%) were involved in the development of every MMI. Only one service answered that they were delivering their MMI screening tool virtually. None of the services indicated that they had attempted to measure either the validity or reliability of the SJT or MMI they were using.

**Table 2:** Comparison of Characteristics with SJT and MMI Use The demographic characteristics of services indicated that they were using SJTs or MMIs when responding to our survey. *Respondents could select multiple answers. MMI, Multiple Mini Interview; SJT, Situational Judgment Test

Characteristic	Using SJTs n (%) (n = 3)	Using MMIs n (%) (n = 4)
Paramedics hired in the past year		
Under 30	2 (66.7%)	3 (75%)
Did not answer	1 (33.3%)	1 (25%)
Province of origin		
Alberta	2 (66.7%)	0
Ontario	0^†^	3 (75%)
Did not answer	1 (33.3%)	1 (25%)
Developers of hiring process*		
Management	3 (100%)	4 (100%)
Human resources	0	4 (100%)
Training department	0	2 (50%)
External consultants	2 (66.7%)	0
Educational institution	1 (33.3%)	0
Amount of time using SJT/MMI		
<2 years	1	2
2-5 years	0	2
11+ years	1	0
Did not answer	1	0

Barriers to SJT and MMI use

The leading reported barriers to the use of SJT and/or MMI in paramedic screening were (Table 3): lack of awareness of these tools (SJT: six (37.5%), MMI: five (35.7%)) and inadequate funding or resources for development of the screening process (SJT: three (18.8%), MMI: three (21.4%)).

**Table 3:** Barriers to Implementing an SJT and/or MMI in the Hiring Process The frequency with which services not using SJTs or MMIs selected that certain barriers were impacting their ability to implement an SJT or MMI. Respondents could select multiple answers. “n” indicates the number of respondents who answered the question. MMI, Multiple Mini Interview; SJT, Situational Judgment Test

Barrier	SJT frequency n (%) (n = 16)	MMI frequency n (%) (n = 14)
Lack of awareness regarding SJTs/MMIs	6 (37.5%)	5 (35.7%)
Lack of funding/resources	3 (18.8%)	3 (21.4%)
Hiring process too small to warrant an SJT/MMI	2 (12.5%)	2 (14.3%)
Lack of reliable research on SJTs/MMIs	2 (12.5%)	1 (7.1%)
The tool seemed redundant with another tool we use	1 (6.3%)	0
A written SJT seemed redundant with our MMI	1 (6.3%)	N/A
A MMI seemed redundant with our written SJT	N/A	0
Unsure	7 (43.8%)	6 (37.5%)
Other	2 (12.5%)	2 (14.3%)

## Discussion

Interpretation

Only a small fraction of respondents used SJTs for paramedic selection; it appears that SJTs have not been broadly adapted for use in paramedic hiring practices in Canada. The leading barriers to the use of SJTs or MMIs were a lack of awareness of them and a lack of resources to develop the screening process, though respondents were most often unsure. The few SJTs that are being used have not been objectively evaluated by the services using them. Only smaller services were using SJTs, despite SJTs being at their most useful in mass-testing [9]. 

Key barriers to wider SJT use in medicine include large upfront costs, the complexity of valid SJT development processes, and growing SJT coaching programs [6,15]. Large upfront costs may be a key, ongoing barrier in the paramedic field, and could be represented by "lack of resources" on our survey. For the development process of an SJT to be theoretically valid, it must be objectively evaluated for construct validity, which was not done by the services in our study [5]. However, the amount of services selecting that they were unsure what barriers were preventing them from using SJTs or MMIs, in addition to the amount of services that selected that awareness of SJTs was a key barrier to their implementation, may suggest that a lack of awareness is the main current barrier to their further use. 

It is interesting to correlate “lack of awareness” as a barrier to SJT and MMI use with the geography of services using SJTs and MMIs within Canada. All services using SJTs were in Alberta; all services using MMIs were in Ontario. Of the four services surveyed in Ontario, three were using MMIs; of the five services surveyed in Alberta, two were using SJTs. No surveyed service was using an MMI or SJT anywhere else in the country. These “pockets” of hiring tool use could also indicate that a lack of awareness is a key barrier, as having a neighboring service adopt a hiring tool could boost awareness. 

We hypothesized that larger services would be more likely to use MMIs and SJTs, as both of these tools are most effective when used for mass testing [9,11]. This hypothesis was not supported by our results; however, it is essential to note that our sample size was especially small in terms of larger paramedic services (only five of the 21 services surveyed were hiring over 80 paramedics per year). All services that were using either SJTs or MMIs and who responded to the demographic questions chose the smallest option available in terms of both the number of employees (less than 200) and paramedics hired per year (less than 30). The services using SJTs had, in addition, been using their SJT for an extended period (two to five years and 11+ years). 

We also hypothesized that services would be testing the reliability and validity of their SJTs and MMIs, but not a single service was doing so. Testing the reliability of either of these tools is key to understanding not only how effective its measurements are but what exactly it is measuring [4]. This vital step being skipped may reflect a lack of resources devoted to personnel selection or a lack of awareness of the research surrounding SJTs and MMIs. 

Implications in practice and research

This survey study was a first step in gaining an understanding of the use of SJT tools for screening paramedic applicants in Canadian paramedic services. No prior paramedic-specific research was found with conclusions on the validity and effectiveness of SJTs as screening tools and on the ideal method in which SJT assessment should be delivered. Additional research is required on the cost-effectiveness of SJTs compared to traditional interviews and other screening methods.

We hypothesized that no service would use both an SJT and an MMI, which was supported by our results. Services could, in theory, use both a written SJT and an MMI, but may prefer to allocate resources toward one or the other due to overlaps in their identified roles as tools for the mass-testing of personal and cognitive skills [10,11]. While a written SJT may be more cost-effective in mass-testing, MMIs may be more valid in evaluating constructs such as NTS [12]. In addition, MMIs have proven valid in the paramedic context, while SJTs are still entirely theoretical in the paramedic context [9,11]. If written SJTs are not as cost-effective when applied to smaller selection processes, smaller paramedic services may find more benefit in using MMIs in the same role. However, SJTs do present unique benefits in terms of improving the diversity of the paramedic workforce due to their written and anonymous nature. Differences in SJT performance, when stratified by race and gender, do exist; these differences are small and may be due to results being confounded by socioeconomic status [2]. The paramedic workforce lacks diversity; SJTs as a selection tool could help ensure selection processes are fair to all demographics of applicants [16]. 

Strengths and limitations

Our survey was the first to attempt to characterize the use of SJTs in paramedic services. We limited our survey to Canadian paramedic services, and the use of SJTs may be more prevalent in other countries. Overall, the response rate was low (21 of 81 services (25.9%)), and this may have been related to the timing of distribution (during the Christmas holiday season). Unanswered demographic questions and “unsure” responses further limited conclusions that could be drawn from the survey, with "unsure" responses specifically limiting the barrier question, as "unsure" was more commonly selected than any barrier. We only recruited through one organization, did not use other recruitment approaches, and only distributed the survey in English; this may have limited the number of paramedic services that participated and may have biased the type of services that were contacted. Our survey was not constructed to learn about SJTs used in contexts other than frontline hiring, such as continuing education. It is possible that the most knowledgeable respondent within a service may not have contributed to survey completion. Our survey might have been made more valid by being piloted on participants with paramedic hiring expertise or on stakeholders in paramedic organizations, rather than students. 

Services most commonly selected “lack of awareness” when asked what barriers prevented their implementation of an MMI or SJT. This answer could be construed as a lack of knowledge within services about the existence of these tools or as a lack of understanding among management of how these tools can help select better applicants. Even if these two possible answers had been separated, there may still have been too many "unsure" responses to draw proper conclusions from this part of the data. Even so, this study provides a pan-Canadian perspective on the use of SJTs and MMIs in hiring practices and offers insights into where research efforts should be directed in the future. 

## Conclusions

Though a role has been identified for SJTs in paramedic selection and education, paramedic services in Canada do not appear to widely use SJTs to inform their hiring processes. Awareness of SJTs and MMIs may be low within paramedic leadership in Canada, but this study was limited in its ability to assess barriers to SJT and MMI use. Similar, more widespread studies should further investigate what barriers are preventing services from using SJTs and MMIs and should aim to identify what types of paramedic services can benefit most from using SJTs. 

## Appendices

The full content of the survey is displayed in tables and divided into groups in the survey logic.

**Table 4:** "Question 1" Group This question acted as the base for the logic of the entire survey, and was considered its own group. Respondents could select multiple answers.

Question	Answer
Which of the following tools does your organization use to screen paramedic applicants?	Interviews
	Clinical scenario test
	Written clinical test
	Multiple mini-interviews (station-based non-clinical interviews)
	Situational judgment test (scenario-based personal/cognitive tests)
	Other (please describe):

**Table 5:** "No MMI/SJT" Group These questions were asked to respondents who indicated that they were not using MMIs or SJTs when asked in question 1. ^*^Respondents could select multiple answers. ^†^Respondents were only asked this question if they responded “Yes” to the previous question. MMI, Multiple Mini Interview; SJT, Situational Judgment Test

Question	Answer
Who designed the hiring process that your organization uses?*	Management
	Human resources
	Clinical training department
	Internal focus group
	External consultants
	Unsure
	Other (please describe):
How long ago did your organization last significantly revise your hiring process?	<2 years
	2-5 years
	6-10 years
	11+ years
	Unsure
Does your organization objectively evaluate its hiring process? (e.g., collection and analysis of outcome data)^†^	Yes
	No
Please describe how your organization has objectively evaluated its hiring process.	(Open ended)
Has your organization considered implementing any personnel selection tools into the hiring process?*	Situational Judgement Test
	Multiple Mini Interview
	No
	Unsure
	Another tool (please describe):

**Table 6:** "SJT" Group These questions were asked to respondents who indicated that they were using SJTs when asked in question 1. ^*^Respondents could select multiple answers. ^†^Respondents were only asked this question if they responded “Yes” to the previous question. MMI, Multiple Mini Interview

Question	Answer
Who designed the Situational Judgement Test (SJT) that you use?*^, †^	Management
	Human resources
	Clinical training department
	Internal focus group
	External consultants
	University
	Other (please describe):
How long have you been using your SJT?	<2 years
	2-5 years
	6-10 years
	11+ years
	Unsure
How does your organization administer your SJT?*	Virtually
	In person
	As part of an MMI
	Other (please describe):
Did your organization attempt to measure the construct validity of your SJT? (i.e., did your organization attempt to measure how well the SJT measures what it is intended to measure)	Yes
	No
	Unsure
Please describe how your organization measured the construct validity of your SJT.^†^	(Open ended)
Did your organization attempt to measure the reliability of your SJT? (i.e., how likely is the same applicant to get the same score when completing the same test at another time)	Yes
	No
	Unsure
Please describe how your organization measured the reliability of your SJT.^†^	(Open ended)
Did your organization assess the performance of the SJT you use in any other ways?*	Qualitative analysis (e.g., exit survey for candidates)
	Quantitative analysis (e.g., collection and analysis of outcome data)
	No
	Unsure
	Other (please describe):

**Table 7:** "MMI" Group These questions were asked to respondents who indicated that they were using MMIs when asked in question 1. ^*^Respondents could select multiple answers. ^†^Respondents were only asked this question if they responded “Yes” to the previous question. ^††^Respondents were only asked this question if they selected that they were using both an MMI and an SJT on question 1. SJT, Situational Judgment Test

Question	Answer
Who designed the Multiple Mini Interview (MMI) that you use?*	Management
	Human resources
	Clinical training department
	Internal focus group
	External consultants
	University
	Other (please describe):
How long have you been using your MMI?	<2 years
	2-5 years
	6-10 years
	11+ years
	Unsure
How does your organization administer your MMI?	Virtually
	In person
	Other (please describe)
Did your organization attempt to measure the construct validity of your MMI? (i.e., did your organization attempt to measure how well the MMI measures what it is intended to measure)	Yes
	No
	Unsure
Please describe how your organization measured the construct validity of your MMI.^†^	(Open ended)
Did your organization attempt to measure the reliability of your MMI? (i.e., how likely is the same applicant to get the same score when completing the same test at another time)	Yes
	No
	Unsure
Please describe how your organization measured the reliability of your MMI.^†^	(Open ended)
Does your MMI include any SJT questions? (scenario-based questions evaluating cognitive/personality traits)^††^	Yes
	No
Do you use a written SJT in addition to your MMI?^††^	Yes
	No

**Table 8:** "Barriers and Demographic Questions" Group Questions from this group were asked to all participants. ^*^Respondents could select multiple answers. ^†^Respondents were only asked this question if they did not indicate that they used SJTs on question 1. ^††^Respondents were only asked the following question if they chose this answer. ^¶^Respondents were only asked this question if they did not indicate that they used MMIs on question 1. MMI, Multiple Mini Interview; SJT, Situational Judgment Test

Question	Answer
Why didn’t your organization implement a written SJT?*^†^	Lack of funding
	Lack of awareness regarding SJTs
	Lack of reliable research on SJTs
	Hiring process too small to warrant the use of an SJT
	A written SJT seemed redundant with our MMI
	A written SJT seemed redundant with another tool we use^††^
	Unsure
	Other (please describe):
What tool made a written SJT seem redundant? (Please describe)	(Open ended)
Why didn’t your organization implement an MMI?*^¶^	Lack of funding
	Lack of awareness regarding MMIs
	Lack of reliable research on MMIs
	Hiring process too small to warrant the use of an MMI
	An MMI seemed redundant with our written SJT
	An MMI seemed redundant with another tool we use^††^
	Unsure
	Other (please describe):
What tool made a MMI seem redundant? (Please describe)	(Open ended)
In what province/territory is your paramedic service located?	Alberta
	British Columbia
	Manitoba
	New Brunswick
	Newfoundland and Labrador
	Northwest Territories
	Nova Scotia
	Nunavut
	Ontario
	Prince Edward Island
	Quebec
	Saskatchewan
	Yukon
What paramedic service do you work for?	(Open ended)
How many paramedics does your service employ?	Less than 200
	200-600
	600-1500
	1500+
How many paramedics have you hired in the past year?	Under 30
	30-80
	80-150
	150+
